# Sleep Apnea Detection with Polysomnography and Depth Sensors

**DOI:** 10.3390/s20051360

**Published:** 2020-03-02

**Authors:** Martin Schätz, Aleš Procházka, Jiří Kuchyňka, Oldřich Vyšata

**Affiliations:** 1 Department of Computing and Control Engineering, University of Chemistry and Technology in Prague, 166 28 Prague 6, Czech Republic; A.Prochazka@ieee.org (A.P.); VysataO@lfhk.cuni.cz (O.V.); 2 Czech Institute of Informatics, Robotics and Cybernetics, Czech Technical University in Prague, 160 00 Prague 6, Czech Republic; 3 University Hospital Hradec Králové, Faculty of Medicine in Hradec Králové, Department of Neurology, Charles University, 500 05 Hradec Králové, Czech Republic; kuchynkajir@seznam.cz

**Keywords:** image processing, signal processing, depth sensors, computational intelligence, human-machine interaction, breathing analysis

## Abstract

This paper is devoted to proving two goals, to show that various depth sensors can be used to record breathing rate with the same accuracy as contact sensors used in polysomnography (PSG), in addition to proving that breathing signals from depth sensors have the same sensitivity to breathing changes as in PSG records. The breathing signal from depth sensors can be used for classification of sleep apnea events with the same success rate as with PSG data. The recent development of computational technologies has led to a big leap in the usability of range imaging sensors. New depth sensors are smaller, have a higher sampling rate, with better resolution, and have bigger precision. They are widely used for computer vision in robotics, but they can be used as non-contact and non-invasive systems for monitoring breathing and its features. The breathing rate can be easily represented as the frequency of a recorded signal. All tested depth sensors (MS Kinect v2, RealSense SR300, R200, D415 and D435) are capable of recording depth data with enough precision in depth sensing and sampling frequency in time (20–35 frames per second (FPS)) to capture breathing rate. The spectral analysis shows a breathing rate between 0.2 Hz and 0.33 Hz, which corresponds to the breathing rate of an adult person during sleep. To test the quality of breathing signal processed by the proposed workflow, a neural network classifier (simple competitive NN) was trained on a set of 57 whole night polysomnographic records with a classification of sleep apneas by a sleep specialist. The resulting classifier can mark all apnea events with 100% accuracy when compared to the classification of a sleep specialist, which is useful to estimate the number of events per hour. When compared to the classification of polysomnographic breathing signal segments by a sleep specialist, which is used for calculating length of the event, the classifier has an $F_{1}$ score of 92.2% Accuracy of 96.8% (sensitivity 89.1% and specificity 98.8%). The classifier also proves successful when tested on breathing signals from MS Kinect v2 and RealSense R200 with simulated sleep apnea events. The whole process can be fully automatic after implementation of automatic chest area segmentation of depth data.

## 1. Introduction

Recently, developed computational technologies have led to a big leap in the usability of range imaging devices. The mentioned depth sensors are smaller, have a higher sampling rate and resolution, but are also much more precise. They are mainly used for computer vision in robotics, and can work as motion controllers for computers and other devices or 3D scanners. In addition, they can be used as non-contact and non-invasive systems for monitoring breathing and its features. The first widely available depth sensor was MS Kinect for Xbox 360 and MS Kinect for Xbox One, which was both successfully tested for complex monitoring of selected biomedical features [1,2,3,4,5,6,7]. They formed an inexpensive replacement for conventional devices. These sensors can be used to monitor breathing and possibly heart rate changes during physical activities or sleep to analyze disorders by mapping chest movements during breathing.

In 2013, Intel presented at a Consumer Electronics Show that it would start focusing on adding motion and voice controls to its computers under the label *perceptual computing*. As such, the technology was re-revealed in 2014 as RealSense (RS) [8]. After research and implementation of said perceptual computing abilities in devices, Intel launched on January 2018 a new Intel RealSense D400 Product Family with Intel RealSense Depth Module D400 Series, and two ready to use depth cameras (Intel RealSense Depth Cameras D435 and D415).

In general, the use of image and depth sensors [1,7,9,10,11,12] allow sleep monitoring and 3D modeling of the chest and abdomen volume changes during breaths as an alternative to conventional spirometry with the use of red-green-blue (RGB) cameras [13,14], infrared cameras [15,16], or Doppler multi-radar systems [17] in addition to ultrasonic and biomotion sensors [18,19,20,21]. The paper provides motivation for further studies of methods related to big data processing problems, dimensionality reduction, principal component analysis, and parallel signal processing to increase the speed, accuracy, and reliability of the results. Methods related to motion recognition can be further used in multichannel biomedical data processing for the detection of neurological disorders, for facial and movement analysis during rehabilitation, and for motion monitoring in assisted living homes [22,23,24].

## 2. Materials and Methods

This work is devoted to proving two goals: showing that various depth sensors can be used to record breathing rate with the same accuracy as contact sensors used in polysomnography (PSG). Section 2.3 is dedicated to spectral analysis of signals recorded by depth sensors and PSG (Table 1), which is used to show the ability of these sensors to capture breathing rate. The next step after preprocessing and comparing signals is to show that breathing signals from depth sensors can be used for classification of sleep apnea events with the same success rate as with PSG data. Section 2.4 is devoted to the classification of sleep apnea events. A simple competitive neural network is trained and tested on a set of PSG data records and successfully tested on a breathing signal recorded by MS Kinect v2 and RealSense R200.

The necessary background is explained in the next subsections.

### 2.1. Data Acquisition

Presented data were recorded either in a sleep laboratory or in a home environment. The overall set consists of 57 patients (demographic overview in Table 2) recorded in a sleep laboratory with polysomnography, where 32 of them were healthy and 25 patients had sleep apnea. To compare the depth sensors properly, an 8-h long set of data was recorded with MS Kinect v2 together with PSG. The PSG has a stable sampling frequency of 10 Hz, and MS Kinect v2 has a mean sampling frequency of 20 Hz. A reason for an unsteady sampling frequency of MS Kinect v2 is that it is not primarily depth sensors, but a device for skeletal data tracking. The sensor is constantly searching for people in space that it would segment and the increased processing demands slows down depth data output. Five other short standalone experimental records of a sleeping person (length 14–43 min.) were recorded with all sensors mentioned in a home environment with the mean sampling frequency being between 30 and 35 Hz. A detailed overview is in Table 1. The stable sampling frequency is necessary to properly capture the motion of a chest during breathing. Each sensor has different recording capabilities and needs different software for recording of data: MS Kinect—C#, Java, Matlab; Intel RS SR300 and R200—Intel Realsense SDK 1.x, C#; Intel RS D415 and D435—Intel Realsense SDK: Librealsense, Viewer (.bag) and MatLab wrapper. The overview of various specifications of depth sensors is in Table 3. The Microsoft Kinect v2 was developed by Microsoft, One Microsoft Way, Redmond, Washington, United States. The Intel RealSense cameras are developed by Intel Corporation, Santa Clara, California, United States.

The sensors included in this study represent a variety of depth sensing devices—from the well-known and widely used MS Kinect v2, which is unique with its time of flight depth sensing technology and its primary function of skeletal tracking to Intel RealSense R200 with its low energy consumption, global shutter, and means to be used in small portable devices. They use tree depth sensing technologies. Time of flight uses IR emitter and camera, the technology counts the time of emitted IR pulse from the device back to the camera, and it recalculates this time to distance. The advantage of this technology is that each pixel of the output depth map has an independent value. Coded light or Structured light technology uses an IR projector and camera. By illuminating scene with a designed static light pattern, at once or by parts, it can determine depth from a single image of reflected light. The disadvantage lies in the design of a pattern, it might mean that, instead of unique depth value for each pixel, the depth might be the same for a few neighboring pixels. Active IR stereo combines the coded Light technology with stereo triangulation. It uses one IR projector and two cameras. The projected light pattern is captured by both cameras and the depth is calculated by estimating disparities between matching points in images from both cameras, resulting in enhanced depth precision.

The ideal candidate for use in this study would be Intel RS D435 with high frames per second (FPS), more precise active IR stereo, and global shutter. However, all mentioned depth sensors are taken into account in testing their ability to capture breathing rate, as there is a reason to use each of them in a different situation, or one cannot choose which to use or acquire.

The MS Kinect v2 was placed 1 meter away from a bed, looking down at the abdomen of a patient at an angle of 45 degrees from a plane of the bed. The recorded patient has a blanket, and it was possible for him to sleep either on his side or on his back despite the restriction of PSG cables and sensors. In a home environment were depth sensors were placed at the head of bed looking down at abdomen of a patient at an angle of 45 degrees from the plane of a bed.

### 2.2. Data Processing

The sequences of depth maps that were acquired by the depth sensors were analyzed over the selected rectangular area with *R* rows and *S* columns that covered the chest area of the individual. For each matrix $D_{n}\left(i,j\right)$ including values inside the region of interest at discrete time *n*, the mean values of a difference between two consequent images were evaluated by the following equation:

(1) $$d\left(n\right)=\frac{1}{R\;S}\sum_{i}^{}\sum_{j}^{}\left(D_{n+1}\left(i,j\right)-D_{n}\left(i,j\right)\right)\;$$

In this way, the mean value of change over each pixel in the selected area for each two depth data forms a separate time series ${\left\{d\left(n\right)\right\}}_{n=0}^{N-2}$ of length N−1.

Then, FIR filtering of the selected order *M* was applied to obtained padded signal ${\left\{d\left(n\right)\right\}}_{n=M-1}^{M+N-3}$ to evaluate the new sequence ${\left\{y\left(n\right)\right\}}_{n=0}^{N-2}$ using the following equation:

(2) $$y\left(n\right)=\sum_{k=0}^{M-1}b\left(k\right)\;d\left(n-k\right)\;$$

with coefficients ${\left\{b\left(k\right)\right\}}_{k=0}^{M-1}$ defined to form a band-pass filter with cut-off frequencies $f_{1}=0.01$ Hz and $f_{2}=1$ Hz to cover the estimated frequency of breathing rate and to reject all other frequency components including the mean signal value and additional high frequency noise. A Savitzky–Golay smoothing filter of the second order might be applied for additional smoothing.

Timestamps recorded with each image frame allowed for resampling of the resulting data with a spline interpolation to a constant sampling rate of $f_{s}=10$ Hz matching sampling rate of PSG.

The resampled data are used for feature extraction and classification using an unsupervised competitive neural network. It is using competitive learning which is in the form of a hidden layer (commonly called a *competitive layer*) where every competitive neuron *i* is described by its vector of weights $w_{i}={\left(w_{i1},\ldots ,w_{id}\right)}^{T},i=1,2,\ldots ,L$ which calculates measure of similarity between the input data $x^{n}={\left(x_{n1},\ldots ,x_{nd}\right)}^{T}\in R^{d}$ and the vector $w_{i}$. The neurons compete each other on every input vector based on greatest similarity to its weight vector. The *l* neuron that wins sets its output $o_{i}=1$ and all other neuron outputs $o_{i}=0,i=1,\ldots ,L,i\neq l$. The inverse of Euclidean distance is usually used as a similarity function together with learning vector quantization (LVQ), where *L* is number of clusters and weight vector $w_{i}={\left(w_{i1},\ldots ,w_{id}\right)}^{T},i=1,2,\ldots ,L$ corresponds to centroid of the cluster *i* [25,26].

Receiver operating characteristics (ROCs) are used for evaluation of the classifier. It is created by a comparison of classification results to target data and by creating a confusion matrix with: true positive (TP) values, equivalent with hit; true negative values (TN), equivalent with correct rejection; false positive (FP), equivalent with Type I error; and false negative (FP), equivalent with Type II error. The measures used in a classifier evaluation are Sensitivity (TPR), Specificity (TNR), Precision (PPV) from Equation (3) and Accuracy (ACC) with $F_{1}$ score from Equation (4). The goal is to have as much accuracy as possible; however, in some cases, $F_{1}$ score is more useful as it considers both the precision and sensitivity:

(3) $$TPR=\frac{TP}{P}=\frac{TP}{TP+FN}\;\;\;TNR=\frac{TN}{N}=\frac{TN}{TN+FP}\;\;\;PPV=\frac{TP}{TP+FP}$$

(4) $$ACC=\frac{TP+TN}{P+N}=\frac{TP+TN}{TP+TN+FP+FN}\;\;\;F_{1}=2\times \frac{PPV\times TPR}{PPV+TPR}=\frac{2TP}{2TP+FP+FN}$$

### 2.3. Breathing Data Processing

To show that the depth sensor records are qualitatively comparable to PSG, various records of breathing during sleep were made with Kinect V2 and RealSense SR300, R200, D415, and D435.

The overall set consists of 57 whole night PSG records, 1 whole night record was made by MS Kinect v2 simultaneously recorded with PSG, and 5 short standalone experimental records with the rest of the sensors mentioned.

**Figure 1:**
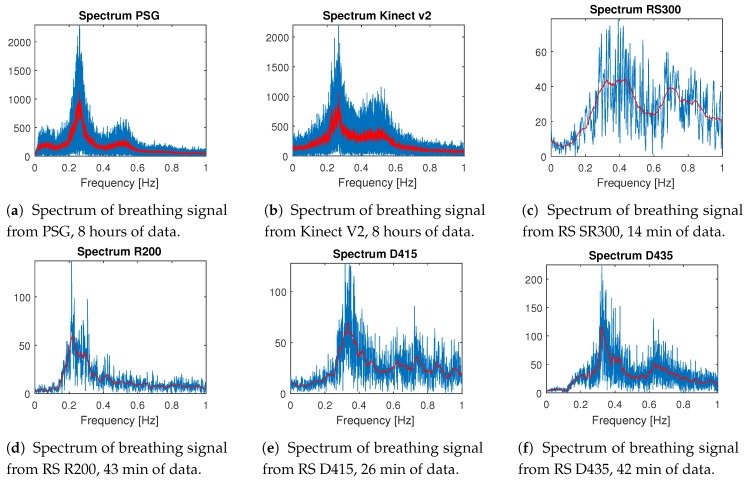
Comparison of spectral analysis of 8 hour records from (**a**) PSG, (**b**) MS Kinect v2 resampled to 10 Hz and (**c**–**f**) shorter signals from RealSense depth sensors.

The most interesting comparison is from whole night sleep data recorded with PSG and MS Kinect v2 at once. The spectrum from one selected PSG record processed with FFT [27] is in Figure 1a and the spectrum from the corresponding MS Kinect v2 record is in Figure 1b. The resting breathing rate of an adult person is between 0.2 and 0.5 Hz while both figures clearly show that both pieces of data have the highest presence of frequency change between 0.2 and 0.3 Hz, with a very similar curve of the breathing data spectrum.

The rest of the records are made in a home environment, one sensor at a time, under the same conditions (angle of view, placement of the sensor, distance from bed), and their details are summarized in Table 1. Except for the MS Kinect v2, all devices have a very stable frame rate around 30 FPS during the whole record. Spectral analysis (Figure 1) from RealSense SR300 is in Figure 1c, RealSense R200 in Figure 1d, RealSense D415 in Figure 1e, and RealSense D435 in Figure 1f. From all these figures, it is clear that all of the sensors can capture the breathing rate of a sleeping person using the described methods, and shows similar trends to the whole night records.

For some of the diagnoses of sleep disorders, it is sufficient to have a breathing rate, but, for diagnosis of sleep apnea, it is very useful to have a breathing signal. The sleep apnea Syndrome [28,29,30,31,32] is characterized by a cessation or decrease of ventilatory effort during sleep and is usually associated with oxygen desaturation [33]. It is divided into three categories: Central Apnea, Obstructive Apnea, and Mixed Apnea. The severity of such syndrome is then measured by a number of episodes during the night and are associated with sleepiness or insomnia. The classification by sleep specialists then usually marks some part of the signal when the events occur because manual marking of all events of 8-hour long records would be unnecessarily time-consuming. However, these data are enough for getting the ground true for classifier training.

### 2.4. Classification of Breathing Data

A classifier from the pilot study [34] was trained on 57 whole night PSG records (32 healthy and 25 patients with sleep apnea) without a teacher based on a competitive neural network works with good accuracy on incomplete classification from a sleep specialist, 94.2% accuracy (vs. sleep specialist). The classifier from the pilot study was trained on features derived from signal energy of the 6th level Haar’s wavelet decomposition. The breathing signal from a depth sensor is different from the one from PSG, mainly because of using a difference of signal in its process. The same approach using unsupervised learning can be used to train a similar classifier on data used in this study.

The strategy to classify a normal breathing signal from that of apnea event is based on analyzing part of a signal of some length. The whole signal is then divided into smaller overlapping pieces. The first feature for the classifier is the most significant frequency. A disadvantage of this feature is that it does not take amplitude of signal into account, so a standard deviation of the signal was selected as a second feature.

Since there are parts of training PSG data that are only partially classified and longer parts of the signal with just one apnea event are rare, it is challenging to create a good training set for supervised learning, which is a reason why unsupervised learning was used. The training set was created from a selected marked apnea event from the set in Table 4. Each segment contains a marked event with 25 s before and after the event so parts of signals with normal breathing were added to the training set. If there were marked events too close to each other (2 central apnea events, 14 obstructive apnea events), the events were not used for training, but they are still useful for evaluation. The circumjacent signal usually contained apnea events missing from original classification These data were used to train two classifiers, simple competitive neural network (2 neurons, 1000 epochs), and K-means classifier with 2 classes. The competitive neural network was selected for further use, as it showed better accuracy (comparison of classifiers statistics is in Table 5).

**Figure 2:**
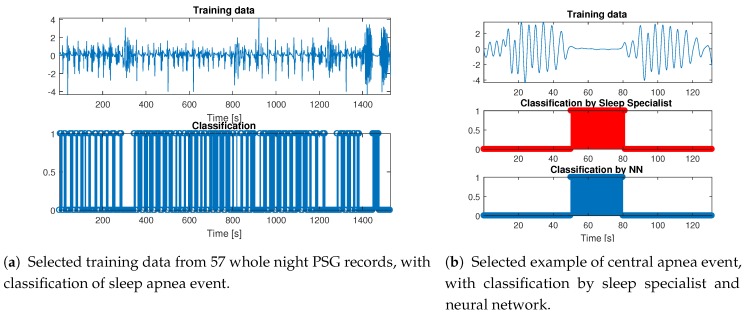
Training data with classification and example of selected central apnea event.

**Figure 3:**
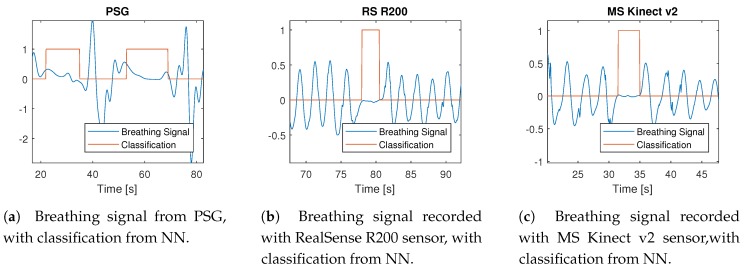
Comparison of sleep apnea classification. All signals are with a sampling frequency of 10 Hz.

The new classifier was successfully tested on data selected from whole night PSG records (Figure 2a), with a selected part with Central apnea events in Figure 3a.

## 3. Results

This paper aims to prove two goals: the first is to show that all mentioned depth sensors can be used to record breathing rate with the same accuracy as contact sensors used in PSG. The second goal is to prove that breathing signals from depth sensors can have the same sensitivity to breathing changes as in PSG data and that it can be used for sleep apnea classification.

The breathing rate can be easily represented as the frequency of a recorded signal. The adult patient should have a breathing rate between 16 and 20 breaths per minute, which is 0.26 Hz and 0.33 Hz. For elderly patients, the breathing rate should not be lower than 10 breaths per minute and higher than 30 breaths per minute, which corresponds to 0.16–0.5 Hz [35,36,37,38,39]. All tested depth sensors are capable of recording depth data with enough precision in depth sensing and sampling frequency (20–35 FPS) in time to capture breathing rate.

To test the quality of breathing data from depth sensors processed by the proposed workflow, a neural network classifier (simple competitive NN) was trained on a set of whole night polysomnographic records with a classification of sleep apneas by a sleep specialist. The training data consist of 57 records containing 32 healthy patients and 25 patients with sleep apnea. Since the original PSG classification by a sleep specialist is done manually, not every apnea event is marked in the classification.

There are two goals for the classification of sleep apnea. The first is to mark correctly that the event occurred, as a number of events per hour or per night is useful criteria for severity ranking of a sleep apnea syndrome. The second goal is to match the classification of a sleep specialist as closely as possible. The length of sleep apnea is also counted in the severity of sleep apnea syndrome [33]. The usual sampling rate of classification used in hospital record is 0.5 seconds, and we decided to use more precise classification with a sampling period of up to 0.1 seconds (10 Hz). The resulting classifier, validated by 5 K-Fold cross-validation with a randomized training set using 70% of segments for training. This classifier can mark all apnea events with 100% accuracy when compared to the PSG classification of a sleep specialist, and with an $F_{1}$ score of 92.2% (Table 5, sensitivity 89.1% and specificity 98.8%) when compared to the classification of breathing signal segments by a sleep specialist (sampling frequency of 10 Hz). The cause of false positive and false negative classification is mainly because of slower change from regular breathing to apnea event. An example of classifier use on one of the segments is in Figure 2b, where there is also graphical comparison to classification by a sleep specialist. Since we can rely on classification from a sleep specialist as ground truth, we can expect that the parts we are unable to compare are classified with the same or similar accuracy.

The classifier was then applied on experimental data with simulated sleep apnea records made with RealSense R200 and MS Kinect V2. These two sensors were selected from all tested devices because they both have highest error estimating distance (MS Kinect V2 [40], 6 mm; RS R200 3 mm [41]). Furthermore, the RS R200 has undefined areas in depth images and works on low power (1.6 W [42]), so it is the best candidate for any further use. The example of successful classification results for RS R200 are in Figure 3b, and the whole record contained four sleep apnea events that the classifier correctly marked. An example of successful results for Kinect v2 is in Figure 3c, and the whole input signal contained five similar sleep apnea events.

## 4. Discussion and Conclusions

The limitation of this study is in the position of the depth sensor and training of the classifier. The processing of depth data can handle a blanket or clothes, but a big distance or bad angle would mean more noise and less recorded movement. The training set could be more balanced with more samples, but as it usually is with biological or biomedical data, it takes time and effort to gather data for a balanced training set. Some of the samples of apnea events are from the same person, but none of the events are repeated in the training set.

Hypothetically, the method can also be used for monitoring newborns with modification of an FIR filter. The breathing rate of newborns is 40–60 breaths per minute, which is 1.33–2 Hz. The proposed method has an upper cut-off frequency at 1 Hz. In theory, a 4 Hz sampling rate would satisfy the Nyquist–Shannon sampling theorem, while most of the sensors have a 30 Hz sampling rate. However, the resolution of depth data should not be a problem, but the movement of the newborn might be problematic.

This paper presents the use of various depth sensors for non-contact monitoring of breathing rate and breathing signal using recorded depth data frames and verification of the obtained results. We showed that the depth sensors MS Kinect v2, Intel RealSense R200, SR300, D415, and D435 are capable of capturing the breathing rate of a sleeping person. The classifier can mark both sleep apnea events and signal segments of sleep apnea events with acceptable accuracy. Both can be used for severity ranking: the first as a number of events per hour and the second for finding out the length of the event. In addition, both types of classification were successfully tested on simulated sleep apnea recorded with MS Kinect v2 and Intel RealSense R200. This method is only a small step before fully automatic use. The future research will be focused on automatic segmentation of a patient’s chest, which is now done manually. Automatic segmentation is so far tested only on data from MS Kinect v2. Quantification of automatic segmentation of a chest area from other depth sensors is planned for the near future.

## Figures and Tables

**Table 1:** Depth data overview.

Depth Sensor	FPS Range	Mean FPS	Length of Data	Sensor Setting
MS Kinect v2	3–29	20	8 h, 19 min	512 × 424 RAW 16bit, 30 FPS
RS SR300	25–45	35	14 min	640 × 480 RAW 16bit, 30 FPS
RS R200	25–43	33	43 min	628 × 468 RAW 16bit, 30 FPS
RS D415	27–33	30	26 min	640 × 480 RAW 16bit, 30 FPS
RS D435	25–37	30	42 min	848 × 480 RAW 16bit, 30 FPS

Frames per second (FPS).

**Table 2:** Demographic overview of patients.

	Healthy		Apnoa	
Sex	Count	Age	Count	Age
Male	21	41.19 ± 15.54	18	49.47 ± 16.65
Female	11	43.09 ± 7.23	7	48.14 ± 12.27

**Table 3:** Comparison of depth sensors.

	Kinect v2	R200	SR300	D415	D435
Depth technology	Time of Flight	Coded Light	Coded Light	Active IR Stereo	Active IR Stereo
Image Sensor tech.	Rolling Shutter	Global Shutter	Global Shutter	Rolling Shutter	Global Shutter
RGB FPS Max	30 FPS	30 FPS	30 FPS, 60 FPS	up to 90 FPS	up to 90 FPS
RGB Resolution	1080p	1080p	1080p, 720p	720p	1080p
Depth Resolution	512 × 424	480p	480p	720p	720p
Depth FPS Max	30 FPS	60 FPS	60 FPS	90 FPS	90 FPS
Range	0.5–4.5 m	0.4–2.8 m	0.2–1.5 m	0.16–10 m	0.11–10 m

Frames per second (FPS).

**Table 4:** Overview of apnea events.

Apnea		Length of Event [s]		
Type	Number of Events	Mean	Min	Max
Central	13	16.2	12.5	31
Obstructive	69	22.8	12.5	37
Mixed	9	21.9	5	44.5

**Table 5:** Results of training set classification, 25.23 min. with Fs of 10 Hz.

Competitive NN				K-Means			
Confusion Matrix		Results		Confusion Matrix		Results	
TP	FP	Sensitivity	89.2%	TP	FP	Sensitivity	9.7%
2855	139	Specificity	98.8%	311	215	Specificity	98.2%
FN	TN	Precision	95.4%	FN	TN	Precision	59.1%
347	11802	$F_{1}$ score	92.2%	2891	11726	$F_{1}$ score	16.7%
